# 4-(Furan-2-carbon­yl)piperazin-1-ium 3,5-di­nitro­benzoate

**DOI:** 10.1107/S160053681401126X

**Published:** 2014-05-24

**Authors:** Channappa N. Kavitha, Manpreet Kaur, Jerry P. Jasinski, Ray J. Butcher, H.S. Yathirajan

**Affiliations:** a Department of Studies in Chemistry, University of Mysore, Manasagangotri, Mysore 570 006, India; b Department of Chemistry, Keene State College, 229 Main Street, Keene, NH 03435-2001, USA; c Department of Chemistry, Howard University, 525 College Street NW, Washington, DC 20059, USA

## Abstract

In the cation of the title salt, C_9_H_13_N_2_O_2_^+^·C_7_H_3_N_2_O_6_^−^, the piperazine ring adopts a slightly distorted chair conformation. Twofold rotational disorder is exhibited by the furan ring in a 0.430 (4):0.570 (4) ratio. In the crystal, N—H⋯O hydrogen bonds link the ions into chains along [010]. Additional weak C—H⋯O inter­actions are observed, leading to a supra­molecular layer parallel to (011).

## Related literature   

For the synthesis of the drug Prazosin {systematic name: 2-[4-(2-furo­yl)piperazin-1-yl]-6,7-di­meth­oxy­quinazolin-4-amine}, see: Honkanen *et al.* (1980 ▶). For the drug 1(2-furo­yl)piperazine, used in the treatment of high blood pressure and anxiety, see: Brogden *et al.* (1977 ▶). For therapeutic uses of piperazines, see: Brockunier *et al.* (2004 ▶); Bogatcheva *et al.* (2006 ▶). For the use of the piperazine moiety in the construction of bioactive mol­ecules, see: Choudhary *et al.* (2006 ▶). For a related structure, see: Dayananda *et al.* (2012 ▶). For puckering parameters, see: Cremer & Pople (1975 ▶).
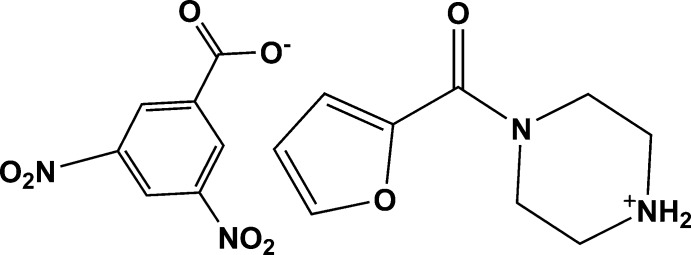


## Experimental   

### 

#### Crystal data  

C_9_H_13_N_2_O_2_^+^·C_7_H_3_N_2_O_6_^−^
*M* *_r_* = 392.33
Orthorhombic, 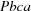

*a* = 9.6060 (2) Å
*b* = 10.4572 (2) Å
*c* = 33.8766 (7) Å
*V* = 3402.97 (13) Å^3^
*Z* = 8
Cu *K*α radiation
μ = 1.08 mm^−1^
*T* = 173 K
0.28 × 0.22 × 0.18 mm

#### Data collection  

Agilent Eos Gemini diffractometer
Absorption correction: multi-scan (*CrysAlis PRO* and *CrysAlis RED*; Agilent, 2012 ▶) *T*_min_ = 0.863, *T*_max_ = 1.000
21195 measured reflections
3352 independent reflections
2915 reflections with *I* > 2σ(*I*)
*R*_int_ = 0.079

#### Refinement  

*R*[*F* ^2^ > 2σ(*F* ^2^)] = 0.043
*wR*(*F* ^2^) = 0.123
*S* = 1.02
3352 reflections
270 parameters
10 restraints
H-atom parameters constrained
Δρ_max_ = 0.26 e Å^−3^
Δρ_min_ = −0.21 e Å^−3^

### 

Data collection: *CrysAlis PRO* (Agilent, 2012 ▶); cell refinement: *CrysAlis PRO*; data reduction: *CrysAlis RED* (Agilent, 2012 ▶); program(s) used to solve structure: *SUPERFLIP* (Palatinus & Chapuis, 2007 ▶); program(s) used to refine structure: *SHELXL2012* (Sheldrick, 2008 ▶); molecular graphics: *OLEX2* (Dolomanov *et al.*, 2009 ▶); software used to prepare material for publication: *OLEX2*.

## Supplementary Material

Crystal structure: contains datablock(s) I. DOI: 10.1107/S160053681401126X/tk5314sup1.cif

Structure factors: contains datablock(s) I. DOI: 10.1107/S160053681401126X/tk5314Isup2.hkl

Supporting information file. DOI: 10.1107/S160053681401126X/tk5314Isup3.cml

CCDC reference: 1003444

Additional supporting information:  crystallographic information; 3D view; checkCIF report

## Figures and Tables

**Table 1:** Hydrogen-bond geometry (Å, °)

*D*—H⋯*A*	*D*—H	H⋯*A*	*D*⋯*A*	*D*—H⋯*A*
N2*B*—H2*BA*⋯O1*A* ^i^	0.99	2.51	3.1607 (16)	123
N2*B*—H2*BA*⋯O2*A* ^i^	0.99	1.72	2.7093 (16)	176
N2*B*—H2*BB*⋯O1*A* ^ii^	0.99	1.77	2.7424 (16)	166
C5*A*—H5*A*⋯O2*A* ^iii^	0.95	2.47	3.3170 (18)	148
C9*B*—H9*B*⋯O6*A* ^iv^	0.95	2.44	3.183 (6)	134
C8*BB*—H8*BB*⋯O3*A* ^v^	0.95	2.50	3.395 (5)	158
C2*B*—H2*BC*⋯O1*B* ^i^	0.99	2.59	3.2300 (19)	122

Symmetry codes: (i) 
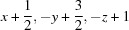
; (ii) 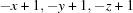; (iii) 
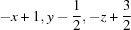
; (iv) 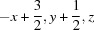; (v) 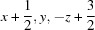.

**Fig. 1.**
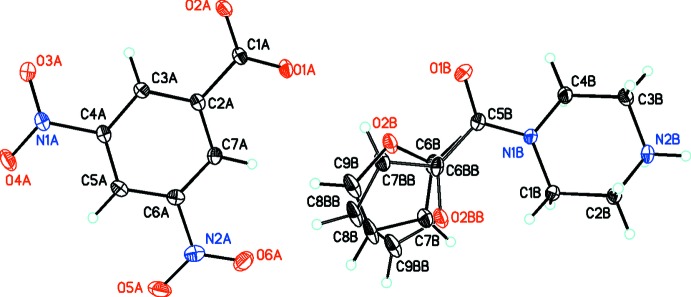
ORTEP drawing of (I) (C9H13N2O2+. C7H3N2O6-) showing the labeling scheme with 30% probability displacement ellipsoids. Two-fold rotational disorder exhibited by the furan ring in a 0.430 (4):0.570 (4) ratio is displayed.

**Fig. 2.**
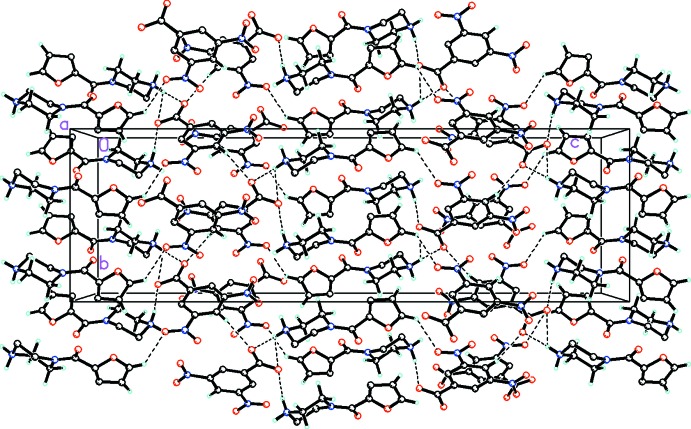
Molecular packing for (I) viewed along the a axis. Dashed lines indicate N—H···O intermolecular hydrogen bonds forming infinite chain along the b axis and weak C—H···O intermolecular interactions. Only the major disordered component [0.570 (4)] of the furan ring is displayed. H atoms not involved in hydrogen bonding have been removed for clarity.

## supplementary crystallographic information

### 2. Structural commentary

1(2-Furoyl)piperazine, used to synthesise the drug Prazosin (Honkanen *et al.*, 1980), is the first of a new class of anti-hypertensives. It is a sympatholytic drug used to treat high blood pressure and anxiety (Brogden *et al.*,1977). Piperazines are found in biologically active compounds across a number of different therapeutic areas (Brockunier *et al.*, 2004; Bogatcheva *et al.*, 2006). The piperazine moiety is extensively employed to construct various bioactive molecules with anti-bacterial and anti-malarial activity, and as anti-psychotic agents (Choudhary *et al.*, 2006). The crystal structures of a similar salt *viz*., cinnarizinium 3,5-di­nitro­salicylate (Dayananda *et al.*, 2012) has been reported. In view of the above importance of piperazines, this paper reports the crystal structure of the title salt, (I) C_9_H_13_N_2_O_2_^+^. C_7_H_3_N_2_O_6_^-^.

The title compound, (I), crystallizes with one independent piperazinium cation and a 3,5-di­nitro­benzoate anion in the asymmetric unit (Fig. 1). In the cation, the piperazine ring adopts a slightly distorted chair conformation with puckering parameters Q, θ, and φ = 0.5552 (15)Å, 173.13 (14)° and 4.2 (14)°, respectively (Cremer & Pople, 1975). Two-fold rotational disorder is exhibited by the furan ring in a 0.430 (4):0.570 (4) ratio represents two different conformations of the molecule that exist in the same crystal form. N—H···O inter­molecular hydrogen bonds link the cations and anions into infinite 1-D chains along [0 1 0] (Fig. 2). Additional weak C—H···O inter­molecular inter­actions are observed (Table 1) forming chains along [0 0 1] resulting in a 2-D supra­molecular network structure.

### 5. Synthesis and crystallization

1(2-Furoyl)piperazine (0.9 g, 0.005 mol) and 3,5-di­nitro­benzoic acid (1.0 g, 0.005 mol) were dissolved in *N*,*N*-di­methyl­formamide and stirred for 10 minutes at 333 K. The resulting solution was allowed to cool slowly at room temperature. The crystals of salt (I) (M.pt: 453–459 K) appeared after a few days.

### 6. Refinement

All of the H atoms were placed in their calculated positions and then refined using the riding model with atom—H lengths of 0.95 Å (CH); 0.99 Å (CH_2_) and 0.92 Å (NH_2_), and with *U*_iso_ = 1.2 x *U*_eq_(parent atom). Owing to poor agreement, the following reflections were omitted from the final cycles of refinement: (2 2 2), (1 2 3), (1 0 6), (0 2 2), (0 4 1), (2 3 0), (1 2 4), (2 1 2), (1 2 1), (2 3 2) and (1 1 13).

### Crystal data

**Table:**

C_9_H_13_N_2_O_2_^+^·C_7_H_3_N_2_O_6_^−^	*D*_x_ = 1.532 Mg m^−^^3^
*M**_r_* = 392.33	Cu *K*α radiation, λ = 1.54184 Å
Orthorhombic, *P**b**c**a*	Cell parameters from 7148 reflections
*a* = 9.6060 (2) Å	θ = 3.9–72.3°
*b* = 10.4572 (2) Å	µ = 1.08 mm^−^^1^
*c* = 33.8766 (7) Å	*T* = 173 K
*V* = 3402.97 (13) Å^3^	Irregular, colourless
*Z* = 8	0.28 × 0.22 × 0.18 mm
*F*(000) = 1632	

### Data collection

**Table:**

Agilent Eos Gemini diffractometer	2915 reflections with *I* > 2σ(*I*)
Detector resolution: 16.0416 pixels mm^-1^	*R*_int_ = 0.079
ω scans	θ_max_ = 72.4°, θ_min_ = 5.2°
Absorption correction: multi-scan (*CrysAlis PRO* and *CrysAlis RED*; Agilent, 2012)	*h* = −10→11
*T*_min_ = 0.863, *T*_max_ = 1.000	*k* = −12→12
21195 measured reflections	*l* = −30→41
3352 independent reflections	

### Refinement

**Table:**

Refinement on *F*^2^	Hydrogen site location: inferred from neighbouring sites
Least-squares matrix: full	H-atom parameters constrained
*R*[*F*^2^ > 2σ(*F*^2^)] = 0.043	*w* = 1/[σ^2^(*F*_o_^2^) + (0.0723*P*)^2^ + 0.8847*P*] where *P* = (*F*_o_^2^ + 2*F*_c_^2^)/3
*wR*(*F*^2^) = 0.123	(Δ/σ)_max_ = 0.001
*S* = 1.02	Δρ_max_ = 0.26 e Å^−^^3^
3352 reflections	Δρ_min_ = −0.21 e Å^−^^3^
270 parameters	Extinction correction: *SHELXL2012* (Sheldrick, 2008), Fc^*^=kFc[1+0.001xFc^2^λ^3^/sin(2θ)]^-1/4^
10 restraints	Extinction coefficient: 0.0015 (3)
Primary atom site location: structure-invariant direct methods	

### Special details

**Table:**

Geometry. All esds (except the esd in the dihedral angle between two l.s. planes) are estimated using the full covariance matrix. The cell esds are taken into account individually in the estimation of esds in distances, angles and torsion angles; correlations between esds in cell parameters are only used when they are defined by crystal symmetry. An approximate (isotropic) treatment of cell esds is used for estimating esds involving l.s. planes.

### Fractional atomic coordinates and isotropic or equivalent isotropic displacement parameters (Å^2^)

**Table:**

	*x*	*y*	*z*	*U*_iso_*/*U*_eq_	Occ. (<1)
O1A	0.36870 (12)	0.57178 (10)	0.63310 (3)	0.0284 (3)	
O2A	0.28703 (12)	0.69735 (10)	0.68122 (3)	0.0317 (3)	
O3A	0.42159 (14)	0.60237 (15)	0.81693 (3)	0.0463 (4)	
O4A	0.58504 (14)	0.46802 (13)	0.82985 (3)	0.0415 (3)	
O5A	0.82825 (13)	0.26144 (13)	0.72229 (4)	0.0456 (3)	
O6A	0.77657 (15)	0.31036 (13)	0.66197 (4)	0.0468 (4)	
N1A	0.51100 (13)	0.52552 (13)	0.80671 (4)	0.0281 (3)	
N2A	0.75838 (14)	0.31806 (13)	0.69759 (4)	0.0326 (3)	
C1A	0.36297 (15)	0.60956 (14)	0.66819 (4)	0.0239 (3)	
C2A	0.45703 (14)	0.54250 (13)	0.69778 (4)	0.0224 (3)	
C3A	0.44092 (15)	0.56475 (13)	0.73799 (4)	0.0223 (3)	
H3A	0.3713	0.6217	0.7473	0.027*	
C4A	0.52856 (15)	0.50207 (14)	0.76415 (4)	0.0233 (3)	
C5A	0.63161 (15)	0.41803 (14)	0.75234 (4)	0.0252 (3)	
H5A	0.6891	0.3744	0.7708	0.030*	
C6A	0.64582 (15)	0.40151 (14)	0.71212 (4)	0.0254 (3)	
C7A	0.56135 (15)	0.46153 (14)	0.68461 (4)	0.0241 (3)	
H7A	0.5748	0.4474	0.6572	0.029*	
O1B	0.49213 (15)	0.72567 (16)	0.51522 (4)	0.0557 (4)	
O2B	0.6605 (4)	0.6627 (4)	0.57013 (17)	0.0347 (6)	0.430 (4)
C6B	0.672 (3)	0.602 (3)	0.5349 (8)	0.023 (2)	0.430 (4)
C7B	0.7724 (9)	0.5082 (8)	0.53851 (19)	0.0311 (8)	0.430 (4)
H7B	0.8108	0.4585	0.5177	0.037*	0.430 (4)
C8B	0.8067 (7)	0.4998 (7)	0.5786 (2)	0.0392 (15)	0.430 (4)
H8B	0.8656	0.4388	0.5910	0.047*	0.430 (4)
C9B	0.7390 (6)	0.5956 (6)	0.59554 (18)	0.0405 (9)	0.430 (4)
H9B	0.7454	0.6149	0.6229	0.049*	0.430 (4)
O2BB	0.7728 (4)	0.5083 (4)	0.52700 (8)	0.0347 (6)	0.570 (4)
C6BB	0.6943 (19)	0.613 (2)	0.5349 (6)	0.023 (2)	0.570 (4)
C7BB	0.6920 (4)	0.6323 (4)	0.57507 (17)	0.0311 (8)	0.570 (4)
H7BB	0.6370	0.6936	0.5887	0.037*	0.570 (4)
C8BB	0.7856 (5)	0.5455 (5)	0.59227 (13)	0.0392 (15)	0.570 (4)
H8BB	0.8115	0.5390	0.6193	0.047*	0.570 (4)
C9BB	0.8307 (4)	0.4732 (4)	0.56174 (14)	0.0405 (9)	0.570 (4)
H9BB	0.8958	0.4054	0.5644	0.049*	0.570 (4)
N1B	0.62643 (12)	0.65199 (13)	0.46545 (3)	0.0251 (3)	
N2B	0.69338 (13)	0.67832 (11)	0.38373 (3)	0.0233 (3)	
H2BA	0.7311	0.7252	0.3607	0.028*	
H2BB	0.6603	0.5938	0.3745	0.028*	
C1B	0.75009 (15)	0.58881 (15)	0.44944 (4)	0.0248 (3)	
H1BA	0.7264	0.5001	0.4417	0.030*	
H1BB	0.8233	0.5849	0.4700	0.030*	
C2B	0.80476 (15)	0.66087 (15)	0.41378 (4)	0.0262 (3)	
H2BC	0.8400	0.7456	0.4222	0.031*	
H2BD	0.8832	0.6128	0.4020	0.031*	
C3B	0.57552 (16)	0.75100 (15)	0.40107 (4)	0.0273 (3)	
H3BA	0.5016	0.7624	0.3810	0.033*	
H3BB	0.6079	0.8367	0.4094	0.033*	
C4B	0.51769 (15)	0.67938 (17)	0.43631 (4)	0.0301 (4)	
H4BA	0.4435	0.7311	0.4488	0.036*	
H4BB	0.4756	0.5980	0.4273	0.036*	
C5B	0.59507 (16)	0.66629 (16)	0.50423 (4)	0.0299 (4)	

### Atomic displacement parameters (Å^2^)

**Table:**

	*U*^11^	*U*^22^	*U*^33^	*U*^12^	*U*^13^	*U*^23^
O1A	0.0419 (6)	0.0242 (5)	0.0189 (5)	0.0007 (4)	−0.0049 (4)	−0.0006 (4)
O2A	0.0430 (6)	0.0275 (6)	0.0246 (5)	0.0102 (5)	−0.0106 (4)	−0.0032 (4)
O3A	0.0509 (8)	0.0651 (9)	0.0228 (6)	0.0221 (7)	0.0003 (5)	−0.0018 (5)
O4A	0.0528 (8)	0.0454 (7)	0.0262 (6)	0.0075 (6)	−0.0137 (5)	0.0072 (5)
O5A	0.0365 (7)	0.0445 (7)	0.0560 (8)	0.0169 (6)	−0.0095 (6)	−0.0018 (6)
O6A	0.0505 (8)	0.0472 (8)	0.0427 (7)	0.0127 (6)	0.0179 (6)	0.0016 (6)
N1A	0.0314 (6)	0.0315 (7)	0.0216 (6)	−0.0008 (5)	−0.0046 (5)	0.0036 (5)
N2A	0.0269 (6)	0.0275 (7)	0.0433 (8)	0.0004 (5)	0.0028 (6)	0.0001 (6)
C1A	0.0305 (7)	0.0198 (7)	0.0214 (7)	−0.0021 (6)	−0.0054 (6)	0.0016 (5)
C2A	0.0249 (7)	0.0206 (7)	0.0218 (7)	−0.0045 (5)	−0.0035 (5)	0.0024 (5)
C3A	0.0233 (7)	0.0204 (7)	0.0233 (7)	−0.0013 (5)	−0.0028 (5)	0.0001 (5)
C4A	0.0252 (7)	0.0240 (7)	0.0207 (6)	−0.0041 (6)	−0.0027 (5)	0.0023 (5)
C5A	0.0234 (7)	0.0235 (7)	0.0287 (7)	−0.0017 (5)	−0.0057 (6)	0.0043 (6)
C6A	0.0223 (7)	0.0224 (7)	0.0316 (7)	−0.0014 (5)	−0.0004 (6)	0.0009 (6)
C7A	0.0268 (7)	0.0238 (7)	0.0215 (7)	−0.0038 (6)	−0.0004 (5)	−0.0007 (5)
O1B	0.0552 (8)	0.0842 (11)	0.0278 (6)	0.0375 (8)	0.0117 (6)	0.0020 (6)
O2B	0.0447 (11)	0.0406 (11)	0.0188 (11)	0.0075 (8)	−0.0056 (12)	0.0038 (13)
C6B	0.019 (5)	0.029 (3)	0.0200 (7)	−0.010 (4)	0.003 (3)	0.0016 (14)
C7B	0.0344 (14)	0.0367 (15)	0.022 (2)	0.0009 (11)	−0.0054 (17)	0.0024 (18)
C8B	0.044 (2)	0.049 (3)	0.024 (2)	−0.016 (3)	−0.017 (2)	0.015 (2)
C9B	0.0447 (16)	0.0482 (18)	0.0284 (14)	−0.0067 (13)	−0.0148 (15)	0.0141 (16)
O2BB	0.0447 (11)	0.0406 (11)	0.0188 (11)	0.0075 (8)	−0.0056 (12)	0.0038 (13)
C6BB	0.019 (5)	0.029 (3)	0.0200 (7)	−0.010 (4)	0.003 (3)	0.0016 (14)
C7BB	0.0344 (14)	0.0367 (15)	0.022 (2)	0.0009 (11)	−0.0054 (17)	0.0024 (18)
C8BB	0.044 (2)	0.049 (3)	0.024 (2)	−0.016 (3)	−0.017 (2)	0.015 (2)
C9BB	0.0447 (16)	0.0482 (18)	0.0284 (14)	−0.0067 (13)	−0.0148 (15)	0.0141 (16)
N1B	0.0236 (6)	0.0343 (7)	0.0174 (6)	0.0045 (5)	0.0012 (4)	0.0017 (5)
N2B	0.0292 (6)	0.0245 (6)	0.0163 (5)	−0.0049 (5)	0.0008 (5)	0.0003 (4)
C1B	0.0267 (7)	0.0294 (7)	0.0184 (6)	0.0052 (6)	0.0015 (5)	−0.0009 (5)
C2B	0.0242 (7)	0.0351 (8)	0.0192 (7)	−0.0006 (6)	0.0010 (5)	−0.0008 (6)
C3B	0.0303 (7)	0.0289 (7)	0.0227 (7)	0.0020 (6)	−0.0026 (6)	0.0030 (6)
C4B	0.0233 (7)	0.0444 (9)	0.0227 (7)	0.0024 (6)	0.0003 (6)	0.0059 (6)
C5B	0.0330 (8)	0.0354 (8)	0.0212 (7)	0.0056 (7)	0.0042 (6)	0.0007 (6)

### Geometric parameters (Å, º)

**Table:**

O1A—C1A	1.2539 (17)	C9B—H9B	0.9500
O2A—C1A	1.2529 (18)	O2BB—C6BB	1.35 (3)
O3A—N1A	1.2261 (18)	O2BB—C9BB	1.352 (5)
O4A—N1A	1.2173 (17)	C6BB—C7BB	1.38 (2)
O5A—N2A	1.2251 (19)	C6BB—C5B	1.517 (9)
O6A—N2A	1.2220 (19)	C7BB—H7BB	0.9500
N1A—C4A	1.4722 (18)	C7BB—C8BB	1.405 (6)
N2A—C6A	1.4741 (19)	C8BB—H8BB	0.9500
C1A—C2A	1.5208 (19)	C8BB—C9BB	1.352 (6)
C2A—C3A	1.3905 (19)	C9BB—H9BB	0.9500
C2A—C7A	1.386 (2)	N1B—C1B	1.4634 (18)
C3A—H3A	0.9500	N1B—C4B	1.4656 (18)
C3A—C4A	1.387 (2)	N1B—C5B	1.3563 (19)
C4A—C5A	1.383 (2)	N2B—H2BA	0.9900
C5A—H5A	0.9500	N2B—H2BB	0.9900
C5A—C6A	1.380 (2)	N2B—C2B	1.4880 (18)
C6A—C7A	1.386 (2)	N2B—C3B	1.4848 (19)
C7A—H7A	0.9500	C1B—H1BA	0.9900
O1B—C5B	1.226 (2)	C1B—H1BB	0.9900
O2B—C6B	1.36 (3)	C1B—C2B	1.5178 (19)
O2B—C9B	1.342 (6)	C2B—H2BC	0.9900
C6B—C7B	1.38 (3)	C2B—H2BD	0.9900
C6B—C5B	1.444 (12)	C3B—H3BA	0.9900
C7B—H7B	0.9500	C3B—H3BB	0.9900
C7B—C8B	1.401 (8)	C3B—C4B	1.515 (2)
C8B—H8B	0.9500	C4B—H4BA	0.9900
C8B—C9B	1.325 (9)	C4B—H4BB	0.9900
			
O3A—N1A—C4A	117.76 (12)	C8BB—C7BB—H7BB	126.2
O4A—N1A—O3A	123.46 (13)	C7BB—C8BB—H8BB	127.8
O4A—N1A—C4A	118.78 (13)	C9BB—C8BB—C7BB	104.4 (4)
O5A—N2A—C6A	117.39 (14)	C9BB—C8BB—H8BB	127.8
O6A—N2A—O5A	124.34 (14)	O2BB—C9BB—H9BB	123.7
O6A—N2A—C6A	118.27 (13)	C8BB—C9BB—O2BB	112.5 (4)
O1A—C1A—C2A	116.97 (13)	C8BB—C9BB—H9BB	123.7
O2A—C1A—O1A	126.19 (13)	C1B—N1B—C4B	114.66 (11)
O2A—C1A—C2A	116.84 (12)	C5B—N1B—C1B	126.09 (12)
C3A—C2A—C1A	120.16 (13)	C5B—N1B—C4B	118.21 (12)
C7A—C2A—C1A	119.94 (13)	H2BA—N2B—H2BB	108.2
C7A—C2A—C3A	119.88 (13)	C2B—N2B—H2BA	109.7
C2A—C3A—H3A	120.7	C2B—N2B—H2BB	109.7
C4A—C3A—C2A	118.65 (13)	C3B—N2B—H2BA	109.7
C4A—C3A—H3A	120.7	C3B—N2B—H2BB	109.7
C3A—C4A—N1A	118.53 (13)	C3B—N2B—C2B	109.90 (11)
C5A—C4A—N1A	118.11 (12)	N1B—C1B—H1BA	109.5
C5A—C4A—C3A	123.36 (13)	N1B—C1B—H1BB	109.5
C4A—C5A—H5A	122.1	N1B—C1B—C2B	110.59 (12)
C6A—C5A—C4A	115.85 (13)	H1BA—C1B—H1BB	108.1
C6A—C5A—H5A	122.1	C2B—C1B—H1BA	109.5
C5A—C6A—N2A	118.44 (13)	C2B—C1B—H1BB	109.5
C5A—C6A—C7A	123.33 (14)	N2B—C2B—C1B	110.90 (12)
C7A—C6A—N2A	118.22 (13)	N2B—C2B—H2BC	109.5
C2A—C7A—C6A	118.91 (13)	N2B—C2B—H2BD	109.5
C2A—C7A—H7A	120.5	C1B—C2B—H2BC	109.5
C6A—C7A—H7A	120.5	C1B—C2B—H2BD	109.5
C9B—O2B—C6B	105.8 (9)	H2BC—C2B—H2BD	108.0
O2B—C6B—C7B	108.3 (9)	N2B—C3B—H3BA	109.7
O2B—C6B—C5B	112 (2)	N2B—C3B—H3BB	109.7
C7B—C6B—C5B	139 (3)	N2B—C3B—C4B	109.78 (12)
C6B—C7B—H7B	126.5	H3BA—C3B—H3BB	108.2
C6B—C7B—C8B	107.1 (13)	C4B—C3B—H3BA	109.7
C8B—C7B—H7B	126.5	C4B—C3B—H3BB	109.7
C7B—C8B—H8B	127.6	N1B—C4B—C3B	111.47 (12)
C9B—C8B—C7B	104.9 (5)	N1B—C4B—H4BA	109.3
C9B—C8B—H8B	127.6	N1B—C4B—H4BB	109.3
O2B—C9B—H9B	123.4	C3B—C4B—H4BA	109.3
C8B—C9B—O2B	113.2 (5)	C3B—C4B—H4BB	109.3
C8B—C9B—H9B	123.4	H4BA—C4B—H4BB	108.0
C6BB—O2BB—C9BB	106.0 (7)	O1B—C5B—C6B	115.6 (15)
O2BB—C6BB—C7BB	109.0 (7)	O1B—C5B—C6BB	119.1 (10)
O2BB—C6BB—C5B	120.9 (17)	O1B—C5B—N1B	121.96 (15)
C7BB—C6BB—C5B	127.6 (18)	N1B—C5B—C6B	122.1 (15)
C6BB—C7BB—H7BB	126.2	N1B—C5B—C6BB	118.8 (10)
C6BB—C7BB—C8BB	107.6 (9)		
			
O1A—C1A—C2A—C3A	−169.82 (13)	C7B—C6B—C5B—N1B	−10 (4)
O1A—C1A—C2A—C7A	11.8 (2)	C7B—C8B—C9B—O2B	2.0 (7)
O2A—C1A—C2A—C3A	10.2 (2)	C9B—O2B—C6B—C7B	−8 (2)
O2A—C1A—C2A—C7A	−168.20 (14)	C9B—O2B—C6B—C5B	−178.6 (14)
O3A—N1A—C4A—C3A	−1.9 (2)	O2BB—C6BB—C7BB—C8BB	6.9 (14)
O3A—N1A—C4A—C5A	178.35 (15)	O2BB—C6BB—C5B—O1B	153.1 (11)
O4A—N1A—C4A—C3A	177.96 (14)	O2BB—C6BB—C5B—C6B	85 (17)
O4A—N1A—C4A—C5A	−1.8 (2)	O2BB—C6BB—C5B—N1B	−29.8 (18)
O5A—N2A—C6A—C5A	4.4 (2)	C6BB—O2BB—C9BB—C8BB	4.1 (10)
O5A—N2A—C6A—C7A	−176.94 (14)	C6BB—C7BB—C8BB—C9BB	−4.2 (10)
O6A—N2A—C6A—C5A	−175.20 (14)	C7BB—C6BB—C5B—O1B	−7 (2)
O6A—N2A—C6A—C7A	3.5 (2)	C7BB—C6BB—C5B—C6B	−75 (17)
N1A—C4A—C5A—C6A	−178.63 (12)	C7BB—C6BB—C5B—N1B	170.0 (13)
N2A—C6A—C7A—C2A	−178.59 (13)	C7BB—C8BB—C9BB—O2BB	0.1 (5)
C1A—C2A—C3A—C4A	−179.95 (12)	C9BB—O2BB—C6BB—C7BB	−6.7 (14)
C1A—C2A—C7A—C6A	179.97 (13)	C9BB—O2BB—C6BB—C5B	−170.1 (11)
C2A—C3A—C4A—N1A	−179.82 (12)	N1B—C1B—C2B—N2B	−54.25 (16)
C2A—C3A—C4A—C5A	−0.1 (2)	N2B—C3B—C4B—N1B	55.11 (17)
C3A—C2A—C7A—C6A	1.6 (2)	C1B—N1B—C4B—C3B	−52.13 (18)
C3A—C4A—C5A—C6A	1.6 (2)	C1B—N1B—C5B—O1B	176.66 (17)
C4A—C5A—C6A—N2A	177.02 (13)	C1B—N1B—C5B—C6B	−9.7 (15)
C4A—C5A—C6A—C7A	−1.6 (2)	C1B—N1B—C5B—C6BB	−0.3 (10)
C5A—C6A—C7A—C2A	0.0 (2)	C2B—N2B—C3B—C4B	−59.34 (15)
C7A—C2A—C3A—C4A	−1.6 (2)	C3B—N2B—C2B—C1B	59.48 (15)
O2B—C6B—C7B—C8B	9 (2)	C4B—N1B—C1B—C2B	51.18 (17)
O2B—C6B—C5B—O1B	−29 (2)	C4B—N1B—C5B—O1B	−15.7 (3)
O2B—C6B—C5B—C6BB	87 (17)	C4B—N1B—C5B—C6B	157.9 (14)
O2B—C6B—C5B—N1B	157.2 (12)	C4B—N1B—C5B—C6BB	167.3 (10)
C6B—O2B—C9B—C8B	3.4 (14)	C5B—C6B—C7B—C8B	176 (3)
C6B—C7B—C8B—C9B	−6.6 (14)	C5B—C6BB—C7BB—C8BB	168.9 (14)
C7B—C6B—C5B—O1B	164 (3)	C5B—N1B—C1B—C2B	−140.79 (15)
C7B—C6B—C5B—C6BB	−80 (17)	C5B—N1B—C4B—C3B	138.83 (15)

### Hydrogen-bond geometry (Å, º)

**Table:**

*D*—H···*A*	*D*—H	H···*A*	*D*···*A*	*D*—H···*A*
N2*B*—H2*BA*···O1*A*^i^	0.99	2.51	3.1607 (16)	123
N2*B*—H2*BA*···O2*A*^i^	0.99	1.72	2.7093 (16)	176
N2*B*—H2*BB*···O1*A*^ii^	0.99	1.77	2.7424 (16)	166
C5*A*—H5*A*···O2*A*^iii^	0.95	2.47	3.3170 (18)	148
C9*B*—H9*B*···O6*A*^iv^	0.95	2.44	3.183 (6)	134
C8*BB*—H8*BB*···O3*A*^v^	0.95	2.50	3.395 (5)	158
C2*B*—H2*BC*···O1*B*^i^	0.99	2.59	3.2300 (19)	122

Symmetry codes: (i) *x*+1/2, −*y*+3/2, −*z*+1; (ii) −*x*+1, −*y*+1, −*z*+1; (iii) −*x*+1, *y*−1/2, −*z*+3/2; (iv) −*x*+3/2, *y*+1/2, *z*; (v) *x*+1/2, *y*, −*z*+3/2.
